# Supplementary material for: Hepatitis C Reinfection in People Who Inject Drugs in Resource-Limited Countries: A Systematic Review and Analysis
Source: Int J Environ Res Public Health. 2020 Jul 9;17(14):4951. doi: 10.3390/ijerph17144951 (PMC7400365; doi:10.3390/ijerph17144951)
Supplement: Supplementary file 1 [file ijerph-17-04951-s001.pdf]

Supplementary Materials: Hepatitis C Reinfection in People Who Inject Drugs in Resource-Limited Countries: A Systematic Review and Analysis

Abbe Muller, David Vlahov, Matthew J. Akiyama and Ann Kurth

Table S1. Sample search strategy.

Database	Search Strategy
Medline	exp Recurrence/
	Reinfection.mp.
	Relapse.mp.
	recurrence.mp.
	2 or 3 or 4
	1 or 2 or 3 or 4
	exp Drug Users/
	exp Substance-Related Disorders/
	exp Substance Abuse, Intravenous/
	drug disorder.mp.
	drug-use.mp.
	PWID.mp.
	exp IDU or IVDU or drug depend** or substance use* or substance misuse* or substance abuse* or drug addict* or opioid substitution* or OST or opioid agonist* or OAT or opioid maintenance* or opiate substitution* or opiate maintenance* or opiate agonist* or methadone therap* or methadone treat* or methadone maintenance* or MMT or buprenorphine therap* or buprenorphine treat* or buprenorphine maintenance* or medication-assisted treat* or medication assisted treat* or MAT or opioid treat* or Opiate Substitution Treatment[mh] or Substance Abuse, Intravenous[mh]
	exp Drug Misuse/
	drug misuse.mp.
	7 or 8 or 9 or 10 or 11 or 12 or 13

	exp Hepatitis C/
	exp Hepacivirus/
	hepatitis c.mp.
	HCV.mp.
	hep c.mp.
	15 or 16 or 17 or 18 or 19
	6 and 14 and 20

© 2020 by the authors. Submitted for possible open access publication under the terms and conditions of the Creative Commons Attribution (CC BY) license (http://creativecommons.org/licenses/by/4.0/).
